# Supplementary material for: The impacts of COVID-19 pandemic on public transit demand in the United States
Source: PLoS One. 2020 Nov 18;15(11):e0242476. doi: 10.1371/journal.pone.0242476 (PMC7673535; doi:10.1371/journal.pone.0242476)
Supplement: Attachment — Submitted filename: response_to_reviewers.docx [file pone.0242476.s001.docx]

# 1 Editor’s comments:

When submitting your revision, we need you to address these additional requirements.

## 1.1 Please ensure that your manuscript meets PLOS ONE's style requirements, including those for file naming. The PLOS ONE style templates can be found at

[https://journals.plos.org/plosone/s/file?id=wjVg/PLOSOne_formatting_sample_main_body.pdf](https://urldefense.com/v3/__https:/journals.plos.org/plosone/s/file?id=wjVg*PLOSOne_formatting_sample_main_body.pdf__;Lw!!KGKeukY!kucfxaRme5LxitUh5oRFWfbWbKThYIMPJSo5NhND4z1Uk5sRRVTtOjrKWSj7QRjyxMk$) and

[https://journals.plos.org/plosone/s/file?id=ba62/PLOSOne_formatting_sample_title_authors_affiliations.pdf](https://urldefense.com/v3/__https:/journals.plos.org/plosone/s/file?id=ba62*PLOSOne_formatting_sample_title_authors_affiliations.pdf__;Lw!!KGKeukY!kucfxaRme5LxitUh5oRFWfbWbKThYIMPJSo5NhND4z1Uk5sRRVTtOjrKWSj7y-wtUI0$)

**Response**: We confirm that our submission is consistent with the two templates above.

## 1.2 In ethics statement in the manuscript and in the online submission form, please confirm that only publicly available data have been used in your study, and that no users' personal information have been accessed or collected.

**Response**: We modified the Ethics Statement as follows:

**[Ethics statement]**: None of the data collected or accessed in this study in this study contain individual personal information. The data in this study are, with one exception, public data. There is one proprietary dataset available by request to the third party.

## 1.3 We note that you have stated that you will provide repository information for your data at acceptance. Should your manuscript be accepted for publication, we will hold it until you provide the relevant accession numbers or DOIs necessary to access your data. If you wish to make changes to your Data Availability statement, please describe these changes in your cover letter and we will update your Data Availability statement to reflect the information you provide.

**Response:** We created an archive with a DOI for our open data, and methods. We also include the procedure for requesting the proprietary data. We modified our Data Availability Statement as follows:

**[Data availability]:** The daily transit demand data, COVID19 confirmed case data, census demographic data, Google search trend data, values behind the reported average and graphs, and code are available at <http://dx.doi.org/10.34740/kaggle/ds/687971>. The hourly transit demand data is proprietary. The archive includes instructions for requesting these data from the third party.

## 1.4 Thank you for stating the following in the Financial Disclosure section:

"The author(s) received no specific funding for this work."

We note that one or more of the authors are employed by a commercial company: "Transit App Inc.,"

a) Please provide an amended Funding Statement declaring this commercial affiliation, as well as a statement regarding the Role of Funders in your study. If the funding organization did not play a role in the study design, data collection and analysis, decision to publish, or preparation of the manuscript and only provided financial support in the form of authors' salaries and/or research materials, please review your statements relating to the author contributions, and ensure you have specifically and accurately indicated the role(s) that these authors had in your study. You can update author roles in the Author Contributions section of the online submission form.

Please also include the following statement within your amended Funding Statement.

“The funder provided support in the form of salaries for authors [insert relevant initials], but did not have any additional role in the study design, data collection and analysis, decision to publish, or preparation of the manuscript. The specific roles of these authors are articulated in the ‘author contributions’ section.”

If your commercial affiliation did play a role in your study, please state and explain this role within your updated Funding Statement.

**Response**: We added the suggested statement to the amended funding statement and the cover letter. We confirm that the commercial affiliation of the third author (Transit app Inc.) did not play a role in our study and there has been no significant financial support for this work that could have influence its outcome.

b)  Please also provide an updated Competing Interests Statement declaring this commercial affiliation along with any other relevant declarations relating to employment, consultancy, patents, products in development, or marketed products, etc.

Within your Competing Interests Statement, please confirm that this commercial affiliation does not alter your adherence to all PLOS ONE policies on sharing data and materials by including the following statement: "This does not alter our adherence to PLOS ONE policies on sharing data and materials.” (as detailed online in our guide for authors [http://journals.plos.org/plosone/s/competing-interests](about:blank)) . If this adherence statement is not accurate and there are restrictions on sharing of data and/or materials, please state these. Please note that we cannot proceed with consideration of your article until this information has been declared.

**Response**: We added the suggested statement to the amended Competing Interests Statement. And we confirm that this commercial affiliation does not alter our adherence to all PLOS ONE policies on sharing data and materials.

Please include both an updated Funding Statement and Competing Interests Statement in your cover letter. We will change the online submission form on your behalf.

**Response**: we added both documents in the cover letter. We thank the editor again for the effort.

Please know it is PLOS ONE policy for corresponding authors to declare, on behalf of all authors, all potential competing interests for the purposes of transparency. PLOS defines a competing interest as anything that interferes with, or could reasonably be perceived as interfering with, the full and objective presentation, peer review, editorial decision-making, or publication of research or non-research articles submitted to one of the journals. Competing interests can be financial or non-financial, professional, or personal. Competing interests can arise in relationship to an organization or another person. Please follow this link to our website for more details on competing interests: [http://journals.plos.org/plosone/s/competing-interests](about:blank)

## 1.5 Please amend either the title on the online submission form (via Edit Submission) or the title in the manuscript so that they are identical.

**Response**: We confirm the titles in the paper and the submission form are identical.

## 1.6 Please remove your figures from within your manuscript file, leaving only the individual TIFF/EPS image files, uploaded separately.  These will be automatically included in the reviewers’ PDF.

**Response**: We apologize for the inconvenience. We removed all figures in the manuscript.

## 1.7 Please ensure that you refer to Figure 4 in your text as, if accepted, production will need this reference to link the reader to the figure

**Response**: We fixed the reference. The graph, which is Fig 6 right now, is referred in section “Results – Decay rate” in the current draft.

## 1.8 We note you have included a table to which you do not refer in the text of your manuscript. Please ensure that you refer to Table 1 in your text; if accepted, production will need this reference to link the reader to the Table.

**Response**: We fixed the reference. The table is referred in section “Results – Base values”, “Results – Base values – African American and Female”, and “Results – Base values - Awareness”.

**2. Comments to the Author**

1. Is the manuscript technically sound, and do the data support the conclusions?

The manuscript must describe a technically sound piece of scientific research with data that supports the conclusions. Experiments must have been conducted rigorously, with appropriate controls, replication, and sample sizes. The conclusions must be drawn appropriately based on the data presented.

Reviewer #1: Yes

Reviewer #2: Yes

2. Has the statistical analysis been performed appropriately and rigorously?

Reviewer #1: No

Reviewer #2: Yes

**Response**: we answer reviewer #1’s question 1 and 3 below and added corresponding explanations in the main text.

3. Have the authors made all data underlying the findings in their manuscript fully available?

The [PLOS Data policy](https://urldefense.com/v3/__http:/www.plosone.org/static/policies.action*sharing__;Iw!!KGKeukY!kucfxaRme5LxitUh5oRFWfbWbKThYIMPJSo5NhND4z1Uk5sRRVTtOjrKWSj7fQGGbiY$) requires authors to make all data underlying the findings described in their manuscript fully available without restriction, with rare exception (please refer to the Data Availability Statement in the manuscript PDF file). The data should be provided as part of the manuscript or its supporting information, or deposited to a public repository. For example, in addition to summary statistics, the data points behind means, medians and variance measures should be available. If there are restrictions on publicly sharing data—e.g. participant privacy or use of data from a third party—those must be specified.

Reviewer #1: No

Reviewer #2: Yes

**Response**: we updated the Data Availability Statement and uploaded all data in an open repository and shared the link and instruction to request the third-party data. Please find our response to the editor’s comment 1.2 and 1.3 above.

4. Is the manuscript presented in an intelligible fashion and written in standard English?

PLOS ONE does not copyedit accepted manuscripts, so the language in submitted articles must be clear, correct, and unambiguous. Any typographical or grammatical errors should be corrected at revision, so please note any specific errors here.

Reviewer #1: Yes

Reviewer #2: No

**Response**: we did a careful proofreading and grammar check on the paper. We thank the reviewers again for the efforts. Please find our responses to reviewer 1’s minor comment 4 and reviewer 2’s comment 6 below.

5. Review Comments to the Author

**Reviewer #1:** This paper studied an interesting and timely research question regarding the transit demand change during the COVID-19 pandemic. The authors employed the data from Transit App to capture transit demand and derived various indexes to describe the change patterns. Overall, this study offers timely data analytics to monitor transit demand during COVID-19. However, there are still several notable concerns with this paper. Detailed comments follow:

The methodological contribution of this paper is limited. Most analyses conducted in this study are descriptive, and the whole paper lacks convincing and strict model build and description:

1) The authors employed a logistic function to fit the transit demand data for each transit system. First, the logistic function is quite different from the logistic model, the authors should be careful when describing their methods. Second, I failed to found any results of the logistic functions. The authors should at least give a summary of the fitting accuracy and statistical significance of the logistic functions for different transit systems.

**Response**: Thank you, this is a good suggestion. We made the corresponding changes:

We changed all “logistic model” to “logistic function” in section “Data and methods – Logistic function for daily transit demand change”^[1]^ to avoid confusion.

We added three test to show logistic function’s goodness of fit: R-squared and QQ plots. We added corresponding method explanations in section “Data and methods – Logistic function for daily transit demand change”; we also added a new section to interpret the logistic function fitting results in section “Results – Logistic function fit”.

The median of all model’s R-squared is 0.969 and 110 of 113 systems’ R-squared is larger than 0.9, which indicates very good fit. The curve fitting plot of every system with raw values and fit values can be found in the data repository: <http://dx.doi.org/10.34740/kaggle/ds/687971>, in the folder “/graphs/curve_fitting”. We moreover used Q-Q plots to test the normality of the residuals. The Q-Q plots show that the results show that each system’s actual quantiles are very close to the theoretical normal distribution quantiles (we show some typical plots in Picture 1 below). For all Q-Q plots for all systems, please find the data repository here: <http://dx.doi.org/10.34740/kaggle/ds/687971>, in the folder “/graphs/qqplots”.

We conclude that logistic function can properly fit the transit demand data with very high fitting accuracy.

Picture 1: QQ plots of some transit systems.

2) When modeling the factors related to floor value, some essential variables are missing. For example, the population density, the job density, and the factors related to transit accessibility (for example, the number of transit stations in each city. The data can be derived from OSM POI). The authors should do more literature review regarding the built environment and public transit to understand which covariates are essential.

**Response**: Under normal conditions, modal shift is a longer-term process affected by built environment, such as building area, commercial and service establishments, accessibility, and road density [1,2]. However, COVID19 is a short-term disruption that is driven by the sudden onset of a public health event. Our intent is to explain the declines in public transit use during a pandemic; we chose a parsimonious set of covariates that focus directly on this mechanism.

However, we understand this critique and agree these transit covariates are worth considering. We revised our analysis to assess including normal public transit use covariates. We describe the results below, our reasoning and modifications to the main text.

We considered population density and employment density (employed civilian population 16 years old and over) at the county-equivalent level. However population density and job density are highly correlated with the ratio of working from home, therefore we did not add the two variables to the final model due to multicollinearity. Given the focus of our analysis on transit demand decline during a pandemic, we believe the ratio of work for home industries is a more direct measure. Moreover, among the three factors, ratio of working from home has the highest R-squared. (The revised draft has a new discussion of these covariates, results and our reasoning in the section “Results –> Base values –> Population with non-physical occupations.”)

We also introduced the Transit Score into the model. Transit Score uses an algorithm to summarize transit accessibility and the relative usefulness of transit routes [3]. The results indicate that Transit Score does not explain the pandemic related decline (p-value = 0.1426).

We added more references to the section “Data and methods –> Logistic function for daily transit demand change –> Base value” to moreover justify the section of independent variables. Our results suggest that the usage rate during the pandemic is more related to economic and demographic factors such as race, job, and age composition than other built environment factors. city facilities and amenities would not change within a short period, therefore it will not be a major factor affecting people’s decision.

Our results suggest that COVID’s impact is unique and cannot explained directly by covariates explaining public transit use under normal conditions. We discuss this as a potential future direction in the conclusion of the revised draft.

3) Is the simple linear model appropriate to fit the floor value? Do the data meet the normality assumption? How to handle spatial auto-correlations? The authors should address these issues before using an OLS model.

**Normality**: The Shapiro-Wilk test shows that we cannot reject the normality assumption for the dependent variable (W = 0.99015, p-value = 0.5917). We also mention that the residuals of the model are normally distributed in section “Result – Base value”: Picture 2 shows the four indicators of regression model assessment. The Q-Q plot (top right) shows that the residual generally follows the normality assumption, which is also proven by the model’s F-test p-value. From these results, we can conclude that the model meets the normality assumption. We added these results in section “Results – Base values”, paragraph 2.

Picture 2: four indicators of the model

**Autocorrelation**: we calculated the univariate Moran’s I with a Queen spatial weights matrix. The Moran’s I is 0.24. Based on this fact, we conducted the spatial lag model regression with the same weight. Tab 1 shows the results: the spatial weight item is not significant. This could be because the other independent variables also have spatial autocorrelation effect, therefore the effect is offset for the residual. Therefore, the results do not support using the spatial autoregressive model.

| Variable | Coefficient | Std.Error | z-value | Probability |
| --- | --- | --- | --- | --- |
| Rho (spatial autocorrelation parameter) | 0.212157 | 0.135082 | 1.57058 | 0.11628 |
| Intercept | -0.38639 | 0.214334 | -1.80276 | 0.07143 |
| Ratio of working from home | -0.46978 | 0.131182 | -3.58111 | 0.00034 |
| Ratio of population over 45 years old | -0.00432 | 0.002269 | -1.90279 | 0.05707 |
| Ratio of African American | 0.803973 | 0.257014 | 3.12813 | 0.00176 |
| Coronavirus Google trend | 0.329794 | 0.084944 | 3.88251 | 0.0001 |
| Ratio of people commuting to work | 0.259591 | 0.171689 | 1.51198 | 0.13054 |
| Ratio of households with no vehicles | -0.19679 | 0.19102 | -1.03023 | 0.3029 |

Tab 1: spatial lag model output summary.

**Other assumptions**: Picture 2 (top left) shows the model generally supports the linearity and homoscedasticity assumptions. Table 1 (in the paper) shows that each independent factor’s variance inflation factor (VIF) is very small, which means there is little lingering multicollinearity. Picture 2 (bottom right) also shows that there are no leverage points in the model and data.

We can conclude that OLS model is appropriate to model the base value (floor value in the last draft) with statistically significant results.

4) Why the authors only build a model for floor value, while ignoring the other indexes like cliff and floor points, response intervals, and the decay rate?

**Response**: we considered models for these parameters in the original drafts of our paper. But these were not in the draft submitted to PLOS One for several reasons:

1. The length of paper is a major factor: the submitted draft is ~8300 words.
2. The regression results using the four parameters mentioned were less interesting and informative than the base value model. This could be because the three time-related parameters (cliff, base points, and response intervals) mostly depend on the temporal development of the pandemic, including the testing process and media. These factors could be highly random and non-linear, therefore making them less relevant to the local demography and built environment. In contrast, base value model had a strong message regarding public transit as an essential service in a community.
3. We do not ignore other parameters in the paper. Instead of using a regression model, we chose to focus on their own spatiotemporal patterns and other, more interesting correlation results for the four parameters. For example, in section “Results – Response intervals” to section “Results – Decay rate” we presented the hyperbolic relationship between decay rates and cliff points because of its good fit and theoretical insights.

The visualization part is insufficient also. At least two figures are important but missing. First, a figure of the transit demand varying patterns across the study period. Second, a figure visualizing the observed data versus the fitted data using the logistic function. The indexes like floor value, cliff and floor points, response intervals, the decay rate, can also be annotated in the figures.

**Response**: we thank the reviewer for the good suggestion. We added the two visualizations as Fig 1 and Fig 2 in the current version. However, we cannot annotate decay rate in the Fig 2, which is a rate and cannot be shown directly on the graph.

Some other minor comments:

1) The authors should involve a proofreader to improve writing. Many words are unprofessional and hard to understand. For example, the floor value mostly means the closest integer less than or equal to a given number, rather than the lowest plateau value the authors want to express.

**Response**: We changed the name “floor value” and “floor point” to “base value” and “base point”. We also did a careful proofreading for the paper and corrected grammar and other issues.

2) The holidays should be excluded from the study periods due to the unusual human mobility patterns.

**Response**: the research time span is from February 15^th^ to May; the only national holiday is President’s day (Feb 17^th^). It is not a major holiday that could impact human mobility such as Christmas and New Year’s Day. Moreover, the transit demand data are normalized and adjusted by historical data; therefore, even if the mobility is impacted, the impact is normalized therefore comparable to other normal days.

3) The authors should also report the variables with insignificant P-values in Table 1.

**Response**: we added the two mentioned insignificant factors “ratio of people commuting to work” and “ratio of households with no vehicles” in the Table 1.

4) In Line 353, why does the ratio of female have high multi-collinearity with the ratio of African Americans?

**Response**: this could be because of African Americans’ demographic structure in the studied cities, which is dominantly large cities. Picture 3 shows the scatter point plot between the two factors. However, we cannot and do not aim to confirm the causality based on correlation results.

Ratio of female

| Ratio of African American | female_ratio |
| --- | --- |
| 0.107766 | 0.50893 |
| 0.0815986 | 0.507154 |
| 0.0815986 | 0.507154 |
| 0.147028 | 0.516009 |
| 0.0815986 | 0.507154 |
| 0.224496 | 0.516486 |
| 0.17749 | 0.51441 |
| 0.0888212 | 0.503332 |
| 0.0987164 | 0.511437 |
| 0.273085 | 0.515614 |
| 0.124931 | 0.515623 |
| 0.0628931 | 0.499394 |
| 0.0628931 | 0.499394 |
| 0.222122 | 0.512007 |
| 0.0858886 | 0.511403 |
| 0.235559 | 0.514782 |
| 0.016857 | 0.505587 |
| 0.136469 | 0.514211 |
| 0.136469 | 0.514211 |
| 0.132354 | 0.517872 |
| 0.113533 | 0.512931 |
| 0.0815986 | 0.507154 |
| 0.225071 | 0.506978 |
| 0.46942 | 0.525373 |
| 0.388955 | 0.51876 |
| 0.46942 | 0.525373 |
| 0.0815986 | 0.507154 |
| 0.296142 | 0.523325 |
| 0.0815986 | 0.507154 |
| 0.205541 | 0.518538 |
| 0.0543381 | 0.504596 |
| 0.0815986 | 0.507154 |
| 0.136469 | 0.514211 |
| 0.501818 | 0.518561 |
| 0.236158 | 0.51647 |
| 0.0628931 | 0.499394 |
| 0.0815986 | 0.507154 |
| 0.0815986 | 0.507154 |
| 0.0815986 | 0.507154 |
| 0.114788 | 0.500471 |
| 0.0815986 | 0.507154 |
| 0.0785536 | 0.507851 |
| 0.209805 | 0.509197 |
| 0.0515889 | 0.503019 |
| 0.441386 | 0.515524 |
| 0.224496 | 0.516486 |
| 0.264429 | 0.516175 |
| 0.283714 | 0.525847 |
| 0.235559 | 0.514782 |
| 0.190189 | 0.502817 |
| 0.117682 | 0.512749 |
| 0.240629 | 0.525766 |
| 0.0172974 | 0.506214 |
| 0.17749 | 0.51441 |
| 0.0258534 | 0.489933 |
| 0.46942 | 0.525373 |
| 0.341354 | 0.529191 |
| 0.32572 | 0.526324 |
| 0.148149 | 0.527012 |
| 0.148149 | 0.527012 |
| 0.148149 | 0.527012 |
| 0.183178 | 0.515054 |
| 0.102491 | 0.516086 |
| 0.273661 | 0.517632 |
| 0.0503846 | 0.496817 |
| 0.0521836 | 0.489715 |
| 0.0503846 | 0.496817 |
| 0.134232 | 0.516236 |
| 0.116993 | 0.513915 |
| 0.148149 | 0.527012 |
| 0.0172974 | 0.506214 |
| 0.0835454 | 0.502451 |
| 0.128832 | 0.517258 |
| 0.235559 | 0.514782 |
| 0.17749 | 0.51441 |
| 0.0815986 | 0.507154 |
| 0.422747 | 0.526796 |
| 0.148149 | 0.527012 |
| 0.0628931 | 0.499394 |
| 0.0521836 | 0.489715 |
| 0.166221 | 0.511716 |
| 0.0960423 | 0.513839 |
| 0.0644258 | 0.502289 |
| 0.114788 | 0.500471 |
| 0.0935671 | 0.499308 |
| 0.0233035 | 0.50668 |
| 0.0815986 | 0.507154 |
| 0.00948258 | 0.504221 |
| 0.422747 | 0.526796 |
| 0.422747 | 0.526796 |
| 0.132354 | 0.517872 |
| 0.388955 | 0.51876 |
| 0.257094 | 0.517598 |
| 0.0628931 | 0.499394 |
| 0.0785536 | 0.507851 |
| 0.257094 | 0.517598 |
| 0.21467 | 0.516598 |
| 0.041491 | 0.506391 |
| 0.0967177 | 0.507519 |
| 0.117769 | 0.504384 |
| 0.0237658 | 0.496125 |
| 0.0815986 | 0.507154 |
| 0.0543381 | 0.504596 |
| 0.17749 | 0.51441 |
| 0.117769 | 0.504384 |
| 0.107766 | 0.50893 |
| 0.0183252 | 0.49809 |
| 0.0547002 | 0.505362 |
| 0.0761751 | 0.5067 |
| 0.0935671 | 0.499308 |
| 0.0250016 | 0.495126 |
| 0.0815986 | 0.507154 |
| 0.46942 | 0.525373 |

Picture 3: the scatter point plot between ratio of African American and ratio of female.

**Reviewer #2:** This interesting paper investigates the impacts of the COVID-19 pandemic of public transit ridership across major systems in the US. The main data supporting this research are provided by the Transit app. It is a very timely effort, focusing on an important topic with a strong tie to the society. Methods are adequate. But I have several concerns for the authors to address.

1. Introduction section is not very motivating. For example, why is it important to study the changes in public transit ridership, along with some metrics like the floor value and so on? What knowledge do we gain from this? How can this knowledge be beneficial to the society? The authors could’ve done a better job discussing these points.

**Response**: We did a major rewrite of the Introduction section as follows:

In paragraph 1, we discussed the timely nature of this paper. As the reviewers pointed out, this is a major contribution of this paper.

In paragraph 2, we discussed the necessity to quantify the highly heterogeneous transit demand decline. We also answered the three questions specially:

- **Why**: the COVID19’s impact on public transit systems is especially severe and highly heterogeneous across the space, according to different reports.
- **Knowledge**: scientific analysis of the impacts of an unprecedented pandemic event on public transit.
- **Societal benefit:** our measures and analysis provide important information for future strategic transit planning and administration. (We also discuss specific insights in the concluding section of the main text.)

In paragraph 3, we discuss the necessity to connect the introduced measures with socioeconomic and demographic factors:

- **Why**: understand the decline and hourly demand profile change’s connection with inequality among different social dimensions.
- **Knowledge**: associations with socioeconomic and demographic factors, especially for underprivileged populations;
- **Societal benefit:** our findings could be a key evidence about the linkages between captive riders and transit system. (We further discuss this in the concluding section of the main text - public transit as a social welfare.

In paragraph 4, we discussed that the literature is very scarce and there is an urgent need to address this academic gap. Very few studies provided systematic discussion about the impact of a pandemic on transit systems on the national scale. This could be another major contribution of this paper.

In paragraph 5, we discussed the new data sources and the opportunities they afford for scientific analysis of public transit decline during a pandemic.

Thank you for this comment. We believe the new introduction section makes a stronger, more focused case for our study.

2. My major concern is that there is no lit review in this paper. Without discussing previous studies of relevant scopes, how can we know the research gap and the contributions of this work? It is important to add such a section to back up your ideas.

**Response**: the reasons why we did not include a dedicated literature review section are:

1. PLOS ONE’s requirement. PLOS ONE requires authors to organize the papers as: Introduction + Materials and Methods + Results + Discussion and Conclusions. Please refer to the official guidelines: <https://journals.plos.org/plosone/s/submission-guidelines>.
2. The topic is unprecedented. Before the occurrence of COVID-19, there were very few studies that investigated the impact of a pandemic on the public transit system. This could be because of two reasons: 1) the lack of accessible empirical transit demand/ridership data; 2) there were very few similar widespread new pandemic such as COVID-19 in the United States.

Therefore, instead of dedicating a whole section for the literature review, we made references in the introduction part and in each corresponding section. We introduced two papers based on Taiwan’s SARS and South Korea’s MERS pandemic and their experience about the pandemic impact on the transit systems. We have yet to find any references about the impact of a pandemic on the transit systems in the North America.

3. Variables. The authors should justify why some variables are selected. I am concerned about a few variables. One such variable, for example, is the occupation type factor. As described in lines 168-169, “Information, Financial activities, and professional and business service” were selected and adopted in the model. The assumption, as detailed in lines 164-165 and line 169, is that these types of workers are more likely to work from home during this pandemic and thus areas with more of these workers are more likely to experience a greater hit in ridership. This assumption/assertion is somehow problematic. I think these subgroups are less likely to use public transit but instead rely more on private vehicles before this pandemic. That said, they may not be an important component to the typical ridership. Therefore, looking at communities with higher percentage of these workers for examining sudden ridership change is less convincing.
In addition to the variables already included in the model, I think the number of homeless people should be considered. Homeless people are more likely to take/occupy public transit, especially in large cities like NYC. As this particular subgroup of population reportedly has higher infection risk, the related transit systems may be affected more severely. This can also be related to the awareness factor discussed in the paper.

**Response**:

**Occupation type**. People who work in information, financial activities, and professional and business services are not necessarily less likely to use public transit; this depends on the community. In some communities, choice riders (people who could drive instead of using public transit) is a substantial proportion of public transit ridership. For example, a 2008 customer experience survey on Chicago Transit Authority (CTA) shows that choice riders account for 62% of the respondents [4]. Moreover, other ridership surveys show that higher-income individuals (highly correlated with the four mentioned industries employees, as we discuss in the main text) are a substantial component of transit ridership in some communities. Nationwide, individuals with income of $100000 or more account for 21% of all transit users, and 23% of all US households according to a 2017 survey conducted by the American Public Transportation Association (APTA) [5]; this is not a large discrepancy.

Our empirical results support the assumption. If the privileged “choice riders” are indeed very few, our regression analysis should not indicate a significant association between the /base value and the ratio of choice riders.

**Homelessness**. To test this, we collected the homeless population data from National Alliance to End Homelessness [6] and conducted correlation and regression analysis between the base value (floor value in the original draft) and the factor “Homeless per 10000 people in the general population”. Picture 4 is the scatter point between the base value and the number of homeless people per 10000 people. Although it is statistically significant, the coefficient is less than 0, which means the more homeless people, the fewer people will continue to use transit during the pandemic. This is contradicting to the assumption that the number of homeless people is a significant factor for the transit decline.

Base value

| Homeless per 10000 people | B |
| --- | --- |
| 15 | -0.610362 |
| 48.1 | -0.865471 |
| 60.5 | -0.739278 |
| 60.5 | -0.780791 |
| 60.5 | -0.784853 |
| 25.8 | -0.910374 |
| 20 | -0.754101 |
| 60.5 | -0.809775 |
| 60.5 | -0.733275 |
| 60.5 | -0.763135 |
| 60.5 | -0.637189 |
| 60.5 | -0.736467 |
| 60.5 | -0.718881 |
| 60.5 | -0.74178 |
| 60.5 | -0.734907 |
| 60.5 | -0.672494 |
| 54.4 | -0.914263 |
| 24.2 | -0.752293 |
| 91 | -0.890967 |
| 24.2 | -0.846063 |
| 60.5 | -0.762912 |
| 60.5 | -0.591584 |
| 60.5 | -0.772279 |
| 91 | -0.885395 |
| 60.5 | -0.69995 |
| 19.6 | -0.814629 |
| 60.5 | -0.689005 |
| 79 | -0.868425 |
| 60.5 | -0.701378 |
| 48.1 | -0.972385 |
| 50.1 | -0.820482 |
| 60.5 | -0.820657 |
| 18 | -0.789494 |
| 18 | -0.958725 |
| 8.6 | -0.64295 |
| 8.6 | -0.719503 |
| 8.6 | -0.641629 |
| 8.2 | -0.71142 |
| 8.6 | -0.690377 |
| 8.6 | -0.691629 |
| 92.8 | -0.790507 |
| 92.8 | -0.65792 |
| 92.8 | -0.817982 |
| 92.8 | -0.765258 |
| 12.6 | -0.563816 |
| 12.4 | -0.600252 |
| 12.6 | -0.631864 |
| 12.6 | -0.532863 |
| 11.5 | -0.503641 |
| 12.6 | -0.501179 |
| 64.6 | -0.553619 |
| 45.1 | -0.774016 |
| 19.5 | -0.788371 |
| 19.5 | -0.755945 |
| 19.5 | -0.723471 |
| 13.9 | -0.705587 |
| 10.9 | -0.572801 |
| 89.9 | -0.829204 |
| 89.9 | -0.802967 |
| 29.2 | -0.703985 |
| 29.2 | -0.72743 |
| 11.6 | -0.957021 |
| 5.2 | -0.976175 |
| 5.2 | -1.00368 |
| 27 | -0.702721 |
|  | -0.377478 |
| 34 | -0.66051 |
| 24.8 | -0.722109 |
| 24.8 | -0.650214 |
| 93.6 | -0.694264 |
| 24.4 | -0.815365 |
| 93.6 | -0.707763 |
| 93.6 | -0.680798 |
| 93.6 | -0.711826 |
| 93.6 | -0.766618 |
| 93.6 | -0.700006 |
| 93.6 | -0.723684 |
| 93.6 | -0.731986 |
| 93.6 | -0.767992 |
| 7.3 | -0.591408 |
| 93.6 | -0.684046 |
| 93.6 | -0.737773 |
| 93.6 | -0.754643 |
| 93.6 | -0.707609 |
| 16.6 | -0.931259 |
| 14.6 | -0.623781 |
| 13 | -0.652185 |
| 12.8 | -0.57233 |
| 13.2 | -0.599654 |
| 13.2 | -0.629972 |
| 49.5 | -0.827596 |
| 49.5 | -0.759263 |
| 6.4 | -0.634232 |
| 36.2 | -0.461256 |
| 36.2 | -0.639929 |
| 36.2 | -0.577701 |
| 10 | -0.799403 |
| 3.8 | -0.714004 |
| 28.7 | -0.742059 |
| 18.1 | -0.754672 |
| 12.5 | -0.553392 |
| 6.5 | -0.657087 |
| 14.5 | -0.46871 |
| 16 | -0.732286 |
| 8.8 | -0.676017 |
| 50.1 | -0.872781 |
| 50.1 | -0.88007 |
| 19.9 | -0.748307 |
| 50.1 | -0.82606 |
| 50.1 | -0.791194 |
| 50.1 | -0.741811 |
| 10.7 | -0.902042 |
| 9.3 | -0.572221 |

Picture 4: scatter point plot between base value and homeless per 10000 people.

Meanwhile, several statistics also show that homeless people are not a major component of transit ridership. For example, in the New York City, one of the cities with highest homeless ratio, numerous local news and city data reported that there are 2000 – 3000 homeless people relied on subway system daily [7,8]. NYC subway’s average workday ridership in 2018 was 4,602,905; the average daily ridership from March to May 2020 was 1,094,822. Therefore, the ratio of homeless ridership is not a significant factor in the total transit ridership.

4. Provide more details. Throughout the paper, the authors claimed that the Transit app is a widely used app. The only statements related to this is in lines 99-101—“the app covers over 200 cities around the world with … download on…” This is insufficient to back up the point that it is a widely used app, and thus leading me to question the representativeness of the data. As the study area is the US, so the authors should provide more details about the user coverage and usage stats (ideally some comparisons with other competitors for showing its market share) to define how “widely” it is being used in the US.

**Response**: this is a good question. We added corresponding explanations in section “Data and methods - Data sources – Transit demand”. Here are some statistics that support the Transit app having a large user base and wide coverage:

1. Transit app’s wide coverage.

As we mentioned in the same section, paragraph 3: according to the daily transit demand data, the studied areas include 63 unique metro areas + 7 state-level/cross-county transit systems. Picture 5 shows that the Transit app has a very wide coverage over almost every major city with transit systems across the United States [9].

Picture 5: Spatial distribution of all transit systems included in our analysis.

2. Transit app’s large user group.

Active users is sensitive data for every mobility app company; therefore, we cannot provide exact numbers of market share or monthly active users. However, we consulted the Transit app and requested some statistics about their usage rate. The US average adoption rate is around 8% in November 2019; for some cities that endorse the app, the rate is 17.3%. For some areas, the market penetration can be larger than 50%.

We make a further estimation: according to the ACS 2018 5-year estimates, there are 7,844,593 workers over 16 years old that use transit systems to work in the US. It is also noteworthy that this number is much underestimated, for the ACS data only include workers over 16 years old and over. Therefore, a rough estimate is that the Transit app user group in the US is in the neighborhood of 1 million.

The wide geographic coverage and large user base make the Transit usage data a worthwhile benchmark for comparing transit decline at a national scale, and its social and community covariates. Nevertheless, the sampling frame is unknown, and we recognize in the main text that our results are a first approximation. We also added a discussion in the main text about public transit users who are likely to be not included in the analysis. Since these are likely to be disadvantaged and older populations, our results in these respects are even more striking.

More details about methods/analyses. Section 2 (section “Data and methods”) describes the analyses/methods, but I find it a bit loosely connected. More details should be provided to better connect these steps and help readers get the full picture.

**Response**: we appreciate the suggestion and added these explanations to the paper: first, we added a new paragraph about the logistic function fitting at the start of the section “Data and methods”. We also added an explanatory graph to explain the curve fitting, the definition of base value, and cliff and base value in Fig 2. We believe this better describes the three key parameters. We also added more details about the statistical test and regression model assumptions thanks to reviewer 1’s suggestions. We also mentioned the equation 1 in each paragraph, where we derive all the key parameters (base value, decay rate, and cliff/base point), to make the method section more compact and connected.

I think it would be great if every city in the maps is labeled.

**Response**: we tried to label every city in the map when we were making this graph, however, the graph becomes too busy and less distinguishable. Picture 5 shows an example when we added all labels, compared with the original graph. The labels will block the points and other labels; adding all labels will make the graph even more undistinguishable. We would like to focus on the spatial pattern of the points, but the labels occupy too much space. Meanwhile, the font size of the labels is also optimized (font size = 13); smaller font will be not distinguishable in the normal size graph.

Picture 6: an example graph with all labels and original graph.

5. Figure 1. Why COVID curve (orange) is more prominent than the typical curve (blue)?

**Response**: we apologize for the mistake. We fixed the caption.

6. There are many typos and formatting issues in the paper, making it difficult to read. The language should be improved.

**Response**: we did a careful review of the grammar and wordings, fixed all the format issues according to the PLOS ONE regulations. We appreciate your patience.

**Reference**:

1.	Chen E, Ye Z, Wang C, Zhang W. Discovering the spatio-temporal impacts of built environment on metro ridership using smart card data. Cities. 2019;95: 102359.

2.	Ma X, Zhang J, Ding C, Wang Y. A geographically and temporally weighted regression model to explore the spatiotemporal influence of built environment on transit ridership. Comput Environ Urban Syst. 2018;70: 113–124.

3.	Walk Score. Transit Score® Methodology. 2020 [cited 23 Jul 2020]. Available: https://www.walkscore.com/transit-score-methodology.shtml

4.	Zhao J, Webb V, Shah P. Customer loyalty differences between captive and choice transit riders. Transp Res Rec. 2014;2415: 80–88.

5.	CJI Research Corporation, Clark HM. Who Rides Public Transportation. 2017. Available: https://www.apta.com/wp-content/uploads/Resources/resources/reportsandpublications/Documents/APTA-Who-Rides-Public-Transportation-2017.pdf

6.	National Alliance to End Homelessness. SOH: State and CoC Dashboards. 2020 [cited 26 Jul 2020]. Available: https://endhomelessness.org/homelessness-in-america/homelessness-statistics/state-of-homelessness-dashboards/

7.	Ricciulli V. When the NYC subway is your home—and you’re evicted every night. 2020 [cited 26 Jul 2020]. Available: https://ny.curbed.com/2020/5/18/21258779/nyc-homeless-subway-closures-trains-unsheltered

8.	Guse C. NYC homeless turn to subway during coronavirus crisis. In: New York Daily News [Internet]. 2020 [cited 23 Jul 2020]. Available: https://www.nydailynews.com/coronavirus/ny-coronavirus-homeless-population-subway-fires-mta-20200421-vr26xwjowzce7hrpimfghrdxxy-story.html

9.	Transit app. Transit • Bus & Subway Times. 2020 [cited 4 Feb 2020]. Available: https://play.google.com/store/apps/details?id=com.thetransitapp.droid&hl=en_US

1. To comply with PLOS ONE’s formats ([https://journals.plos.org/plosone/s/file?id=wjVg/PLOSOne_formatting_sample_main_body.pdf](https://urldefense.com/v3/__https:/journals.plos.org/plosone/s/file?id=wjVg*PLOSOne_formatting_sample_main_body.pdf__;Lw!!KGKeukY!kucfxaRme5LxitUh5oRFWfbWbKThYIMPJSo5NhND4z1Uk5sRRVTtOjrKWSj7QRjyxMk$)), therefore we had to remove the multilevel numbers in the section titles.
